# IL-27 Driven Upregulation of Surface HLA-E Expression on Monocytes Inhibits IFN-**γ** Release by Autologous NK Cells

**DOI:** 10.1155/2014/938561

**Published:** 2014-03-10

**Authors:** Fabio Morandi, Irma Airoldi, Vito Pistoia

**Affiliations:** Laboratory of Oncology, Istituto Giannina Gaslini, Via Gaslini 1, 16148 Genova, Italy

## Abstract

HLA-G and HLA-E are HLA-Ib molecules with several immunoregulatory properties. Their cell surface expression can be modulated by different cytokines. Since IL-27 and IL-30 may either stimulate or regulate immune responses, we have here tested whether these cytokines may modulate HLA-G and -E expression and function on human monocytes. Monocytes expressed gp130 and WSX-1, the two chains of IL27 receptor (R), and IL6R*α* (that serves as IL-30R, in combination with gp130). However, only IL27R appeared to be functional, as witnessed by IL-27 driven STAT1/ STAT3 phosphorylation. IL-27, but not IL-30, significantly upregulated HLA-E (but not HLA-G) expression on monocytes. IFN-*γ*; secretion by activated NK cells was dampened when the latter cells were cocultured with IL-27 pretreated autologous monocytes. Such effect was not achieved using untreated or IL-30 pretreated monocytes, thus indicating that IL-27 driven HLA-E upregulation might be involved, possibly through the interaction of this molecule with CD94/NKG2A inhibitory receptor on NK cells. In contrast, cytotoxic granules release by NK cell in response to K562 cells was unaffected in the presence of IL-27 pretreated monocytes. In conclusion, we delineated a novel immunoregulatory function of IL-27 involving HLA-E upregulation on monocytes that might in turn indirectly impair some NK cell functions.

## 1. Introduction

HLA-class Ib family represents a small group of HLA-class I molecules that include HLA-G, HLA-E, HLA-F, and HLA-H [1]. In contrast with highly polymorphic classical HLA-class Ia molecules that are mainly involved in the presentation of peptides recognized by the T cell receptor on T lymphocytes, as well as in the interaction with killer Ig-like receptors on NK cells, HLA-Ib molecules are characterized by a very low degree of polymorphism and display several immunoregulatory properties [2].

HLA-G and -E are the best characterized among HLA-Ib molecules. HLA-G can interact with four inhibitory receptors, namely, immunoglobulin-like transcript (ILT)2, ILT4, KIR2DL4, and CD160, and this interaction leads to the inhibition of immune effector cell functions. The physiological role of HLA-G is to abrogate the maternal NK cell response against fetal tissue at maternal/fetal interface [3].

HLA-E presents peptides derived from the leader sequence of other HLA-class I molecules to NK cells, by interacting with the CD94/NKG2A receptor. When target cells express normal levels of HLA and consequently HLA-derived peptides are generated, this interaction takes place, leading to the inhibition of NK cell lysis. In contrast, cells with low to absent HLA-class I expression (i.e., tumor cells and virus-infected cells) generate low amounts of HLA-derived peptides, and consequently their surface expression of HLA-E is low. The loss of interaction between surface HLA-E and CD94/NKG2A provide a “kill” signal to NK cells [4].

HLA-G expression can be modulated by different cytokines, such as IL-10 [5, 6], IFN-*γ* [7], IFN-*α* [8], IFN-*β* [9], and TGF-*β* [10]. Similarly, modulation of HLA-E expression has been reported by different authors, in response to IFN-*γ* [11–16], TNF-*α*, and IL-1*β* [12]. In this context, a possible modulation of HLA-G and -E expression by IL-27 may be interesting, since both immunostimulatory and immunoregulatory properties have been ascribed to this cytokine [17].

IL-27 belongs to the IL-12 family and is formed by EBI3 (also shared by IL35) and p28 (homologous to p35 and p40 subunits of IL-12) [18]. The IL-27 receptor (R) is composed by WSX-1 (also known as IL-27R*α*/TCCR) and gp130 chains [19]. Signal transduction initiated by IL-27 involves the phosphorylation of STAT molecules, especially STAT-1 and -3, in different immune effector cells [18, 20–22]. Very recently, the IL-27 p28 subunit has been described as an independent cytokine, also known as IL-30, that may function in the absence of the EBI3 subunit [23, 24]. p28 exerts anti-inflammatory effects by acting as antagonist of gp-130 mediated signaling initiated by IL-6 or IL-27 [24]. In addition, IL-30 may signal independently through IL-6R*α*, by recruiting gp130 homodimers [25].

In this paper, we report for the first time the modulation of a member of the HLA-class Ib molecule family by IL-27 in human monocytes, and we provide the first evidence of a differential effect of IL-27 and IL-30 on a specific human immune cell population.

## 2. Materials and Methods

### 2.1. Cell Isolation and Flow Cytometry

Peripheral blood samples were obtained from six different normal donors afferent to the blood bank of Istituto Giannina Gaslini, after written informed consent. Monocytes and NK cells were isolated using RosetteSep Human Monocyte Enrichment Cocktail and RosetteSep Human NK cells Enrichment Cocktail, respectively (StemCell Technologies), following manufacturer's protocol.

The expression of gp130, WSX-1, and IL-6R*α* was evaluated on freshly isolated monocytes using the following monoclonal antibodies (mAbs): FITC-conjugated anti-gp130, PE-conjugated anti-WSX-1, and PE-conjugated anti-IL-6R*α* (R&D Systems). Fluorochrome and isotype-matched irrelevant antibodies (Beckman Coulter) were used as negative control. Cells were stained for 20 min in the dark at 4°C and then washed in PBS (Sigma) supplemented with 1% FBS (Euroclone).

IL-27 and IL-30 driven signal transduction was analyzed on monocytes cultured for 30′ at 37°C and 5% CO_2_ in the presence or absence of human recombinant (hr)IL-27 (R&D System, 100 ng/mL) or hrIL-30 (Abnova, 100 ng/mL), using FITC-conjugated anti-phospho (p)STAT1, anti-pSTAT3, and anti-pERK1/2 mAbs (Cell Signaling) following manufacturer's protocol.

HLA-G and -E expression was evaluated on monocytes cultured for 24 hours at 37°C and 5% CO_2_ in the presence or absence of hrIL-27 (R&D System, 100 ng/mL) or hrIL-30 (Abnova, 100 ng/mL), using purified MEM-G9 (Exbio) and 3D12 (Biolegend) mAbs, respectively. Isotype-matched irrelevant antibody (Beckman Coulter) was used as negative control. Cells were stained for 20 min in the dark at 4°C and washed in PBS (Sigma) supplemented with 1% FBS (Euroclone). Cells were then incubated with PE-conjugated goat anti-mouse IgG1 (Beckman Coulter) as secondary reagent.

Cells were run on Gallios cytometer (Beckman Coulter). 10^4^ events were collected. FACS analysis was performed using Kaluza software (Beckman Coulter). Data were expressed as mean relative of fluorescence intensity (MRFI = mean of fluorescence intensity obtained with specific antibody/mean of fluorescence intensity obtained with irrelevant isotype-matched antibody).

### 2.2. Degranulation Assay

Freshly isolated NK cells (10^5^ cells) were cultured in RPMI-1640 medium (Euroclone) supplemented with 10% FBS (Euroclone) using round-bottom 96-well plates (Corning), in the presence or absence of target cells (K562 cell line) at 1 : 4 effector/target ratio. In some experiments, autologous monocytes (2.5 × 10^4^ cells, pretreated or not with IL-27 or IL-30, as described above) were added as third-party cells. PE-conjugated anti-CD107a mAb (Miltenyi Biotec) was added to each well. Cells were incubated for 5 hours at 37°C and 5% CO_2_. Cells were then washed and run on Gallios cytometer (Beckman Coulter). 10^4^ events were collected. FACS analysis was performed using Kaluza software (Beckman Coulter). Data were expressed as percentage of CD107a^+^ cells, gating on NK cells.

### 2.3. IFN-*γ* Release by NK Cells

Flat-bottom 96-well plates (Corning) were coated overnight at 4°C with 100 *μ*L of anti-NKp46 activating receptor mAb (2.5 *μ*g/mL, Miltenyi Biotec) or PBS. Plates were then washed 3 times in PBS. NK cells were plated at 10^5^ cells/well in RPMI-1640 10% FBS supplemented with IL12p70 (R&D System, 0.1 ng/mL), in the presence or absence of autologous monocytes (2.5 × 10^4^ cells, pretreated or not with IL-27 or IL-30, as described above). Cells were incubated at 37°C and 5% CO_2_ for 24 hours. Supernatants were then collected and centrifuged at 3000 g for 10 minutes. IFN-*γ* was measured using IFN-*γ* ELISA set (Immunotools), following the manufacturer's protocol. Absorbance at 450 nm was measured using Infinite 200 PRO spectrometer (Tecan Group Ltd.). Results are expressed as ng/mL IFN-*γ*.

### 2.4. Statistical Analysis

Statistical analysis was performed using Prism Software (GraphPad Software Inc.). The normality of each variable was checked by using the Kolmogorov-Smirnov test. When normality of data distribution was found in all variables, statistical analysis was performed by a parametric approach. Conversely, when normality of data distribution was rejected in several variables, a nonparametric analysis was applied. Accordingly, *t*-test or Mann-Whitney test was used.

## 3. Results

### 3.1. Human Monocytes Expressed Complete and Functional IL-27R

**Figure 1:**
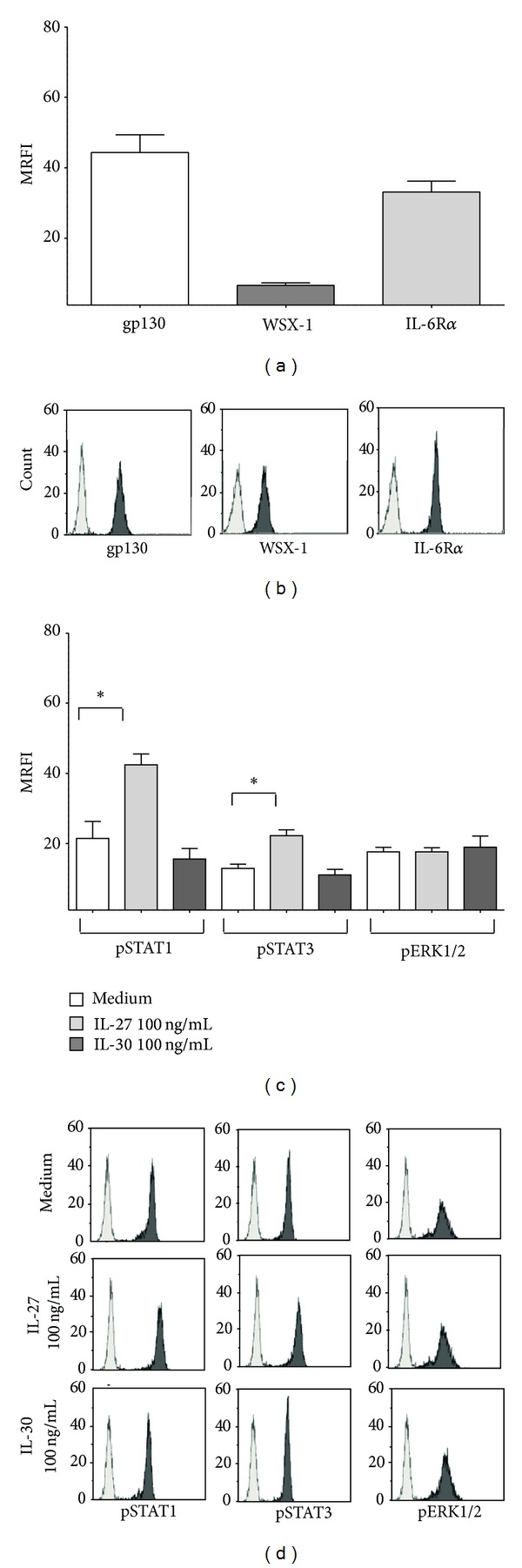
Expression and function of IL27R and IL30R in human monocytes. (a) Expression of gp130 (white bar), WSX-1 (grey bar), and IL6R*α* (light grey bar) was evaluated by flow cytometry in freshly isolated monocytes. Results are represented as MRFI. Histograms represent mean of six different experiments ± SD. One representative FACS analysis is reported in (b). Grey profiles show staining with irrelevant fluorochrome and isotype matched mAb. Black profiles show staining with specific mAb. (c) STAT1, STAT3, and ERK1/2 phosphorylation was investigated in monocytes cultured with medium alone (white bars), 100 ng/mL IL-27 (light grey bars), or 100 ng/mL IL-30 (grey bars). Results are represented as MRFI. Histograms represent mean of six different experiments ± SD. Asterisks indicate statistical significance. One representative FACS analysis is reported in (d). Grey profiles show staining with irrelevant fluorochrome and isotype matched mAb. Black profiles show staining with specific mAb.

The expression of gp130 and WSX-1 (the two subunits of IL-27R) and IL-6R*α* (that serves as receptor for IL-30 in combination with gp130) was tested on freshly isolated human monocytes. As shown in Figure 1(a), monocytes expressed very high levels of gp130 (MRFI ± SD: 44.35 ± 8.69) and IL6R*α* (MRFI ± SD: 33.11 ± 5.37), whereas WSX-1 expression (MRFI ± SD: 6.56 ± 1.33) was lower. A representative FACS analysis is shown in Figure 1(b).

Next, we asked whether IL-27R and IL-30R expressed by human monocytes were functional. As shown in Figure 1(c), IL-27 treatment increased the phosphorylation of STAT1 (MRFI ± SD: medium 21.44 ± 8.32; IL-27 42.45 ± 5.26; *P* = 0.05) and STAT3 (MRFI ± SD: medium 12.85 ± 2.1; IL-27 22.2 ± 2.86; *P* = 0.05), but not of ERK1/2. Conversely, no significant upregulation of pSTAT1, pSTAT3, or pERK1/2 was observed after treatment of monocytes with IL-30. A representative FACS analysis is shown in Figure 1(d).

Thus, taken together, these data suggested that IL27R, but not IL30R, was functional in human monocytes. Another possible explanation is that IL-30 driven signaling may be mediated by the activation of additional pathways that do not include STAT or ERK molecules, as reported in other human immune cells.

### 3.2. IL-27 Treatment Induced Surface HLA-E Upregulation on Monocytes

**Figure 2:**
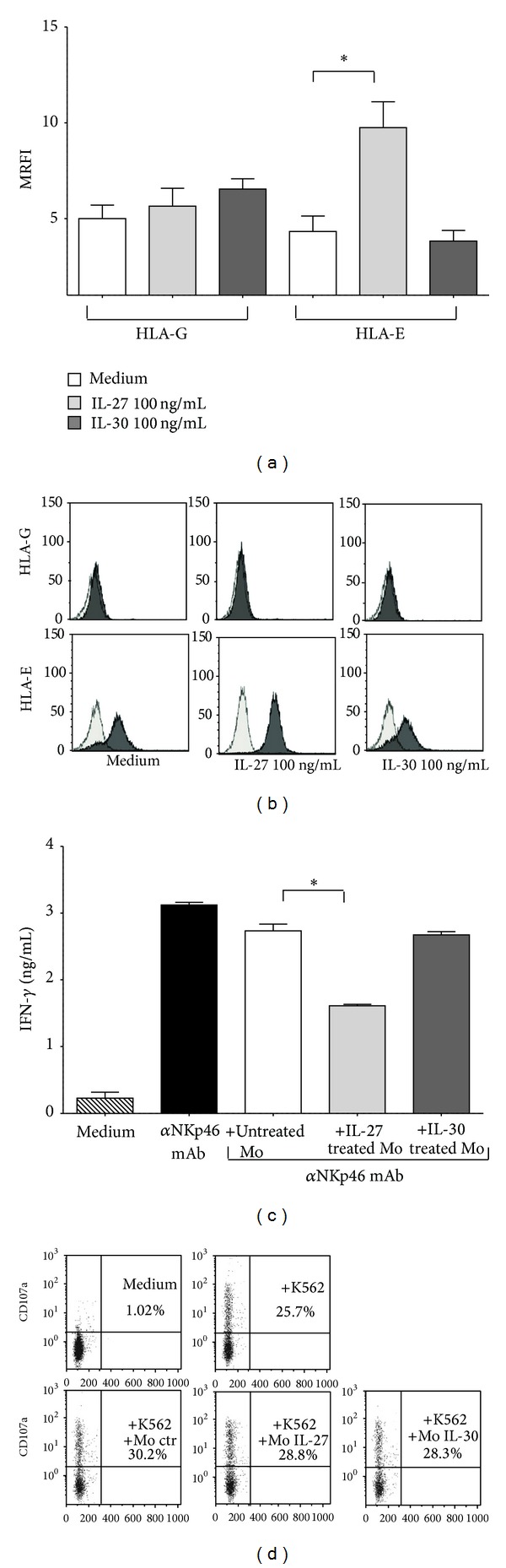
Modulation of HLA-G and HLA-E expression in human monocytes and functional assays on NK cells. (a) HLA-G and -E expression was investigated in monocytes cultured with medium alone (white bars), 100 ng/mL IL-27 (light grey bars), or 100 ng/mL IL-30 (grey bars). Results are represented as MRFI. Histograms represent mean of six different experiments ± SD. Asterisks indicate statistical significance. One representative FACS analysis is reported in (b). Grey profiles show staining with irrelevant fluorochrome and isotype matched mAb. Black profiles show staining with specific mAb. (c) IFN-*γ* was analyzed in supernatants from NK cells cultured with (i) medium alone (stripped bar), (ii) in the presence of coated anti-NKp46 mAb (black bar) or in the presence of coated anti-NKp46 mAb and autologous monocytes, (iii) untreated (white bar), (iv) pretreated with IL-27 (light grey bar), or (v) pretreated with IL-30 (grey bar). Results are represented as ng/mL IFN-*γ*. Histograms represent mean of six different experiments ± SD. Asterisks indicate statistical significance. (d) Expression of CD107a was evaluated on NK cells cultured with (i) medium alone, (ii) target cells (K562) at 4 : 1 effector : target ratio, and target cells (K562) in the presence of autologous monocytes, (iii) untreated, (iv) pretreated with IL-27, or (v) pretreated with IL-30. One representative experiment out of three performed is shown. The percentage of CD107a^+^ cells (gating on NK cells using physical parameters) is indicated.

We have next investigated whether IL-27 or IL-30 may modulate the surface expression of two HLA-class Ib molecules on human monocytes. As shown in Figure 2(a), IL-27 treatment significantly upregulated HLA-E (MRFI ± SD: medium 4.33 ± 1.95; IL-27 9.75 ± 3.31; *P* = 0.01) but not HLA-G expression on monocytes. In contrast, no modulation of HLA-G and -E expression was observed after treatment of monocytes with IL-30. A representative FACS analysis is shown in Figure 2(b).

### 3.3. IL-27 Treated Monocytes Inhibited IFN-*γ* Release by NK Cells but Not Cytotoxicity

Finally, we have investigated whether the IL-27 driven upregulation of HLA-E on monocytes may functionally impair autologous NK cell function. To this end, we have investigated IFN-*γ* secretion and cytotoxic activity of NK cells cultured in the presence or absence of autologous monocytes, previously treated with medium alone, IL-27, or IL-30.

IFN-*γ* secretion was investigated on supernatants from NK cells cultured in different experimental conditions. As shown in Figure 2(c), IFN-*γ* secretion was low to absent in NK cells cultured with medium alone (mean ng/mL ± SD: 0.22 ± 0.18), whereas NK cells cultured in the presence of coated anti-NKp46 agonist mAb secreted high amounts of IFN-*γ* (mean ng/mL ± SD: 3.12 ± 0.08). Such secretion was significantly reduced when NK cells were cocoltured with autologous monocytes pretreated with IL-27 (mean ng/mL ± SD: 1.61 ± 0.04, *P* = 0.01), but not with untreated (mean ng/mL ± SD: 2.73 ± 0.19) or IL-30 pretreated (mean ng/mL ± SD: 2.67 ± 0.1) monocytes.

Cytotoxic activity of NK cells was next assessed by investigating the secretion of lytic granules in response to target cells, witnessed by CD107a expression on the cell surface. NK cells cultured with medium alone displayed a low to absent degranulation (% CD107a^+^ cells ± SD: 0.81 ± 0.29) that was dramatically increased in the presence of K562 cell line (% CD107a^+^ cells ± SD: 29.21 ± 4.95). NK cells preserved the ability to secrete lytic granules in response to K562 cell line when cultured in the presence of autologous monocytes pretreated with IL-27 (% CD107a^+^ cells ± SD: 34.06 ± 7.43), IL-30 (% CD107a^+^ cells ± SD: 30.35 ± 2.77), or medium alone (% CD107a^+^ cells ± SD: 31.47 ± 1.73). A representative FACS analysis is shown in Figure 2(d).

Our results indicated that IL-27 treated monocytes that upregulated surface HLA-E expression were able to dampen IFN-*γ* secretion by activated autologous NK cells, probably through the interaction of HLA-E with the inhibitory receptor CD94/NKG2A expressed on NK cells. Conversely, such interaction was not sufficient to inhibit the release of cytotoxic granules by NK cells in the presence of HLA-class I deficient target cells.

## 4. Discussion

The role of HLA-class Ib molecules in the control of the immune system has been clearly described in the last years [26, 27]. Surface and soluble HLA-G molecules abrogate the function of different immune effector cells, such as NK cells, T cells, and B cells, through different mechanisms [3]. A high expression of HLA-E on the surface of target cells may protect them from NK cell mediated lysis [28], and this feature is commonly used as immune escape mechanism by virus-infected cells [15] or tumor cells [29]. In addition, several authors have demonstrated that cells with high surface HLA-E expression may modulate other NK cell functions through the interaction with CD94/NKG2A on NK cells, for example during trophoblast recognition by decidual NK cells [30].

No information is available regarding a possible modulation of HLA-G and -E expression by IL-27. It has been previously reported that IL-27 exerts a proinflammatory activity on human monocytes, inducing an augmented response to TLR signals [31, 32] and the release of proinflammatory cytokines and chemokines [33] mainly through STAT1, STAT3, and NF-*κ*B activation.

In line with these data, we have here demonstrated that in monocytes IL-27 signals through STAT1 and STAT3 phosphorylation, whereas ERK pathway was unaffected by IL-27 treatment. In addition, we have demonstrated for the first time that IL-30 displays a different behavior, since molecules involved in IL-27 driven signaling pathway were unaffected by IL-30. It is conceivable that additional pathway(s) other than that of STAT and ERK might be involved in IL-30 signaling in monocytes. Another possible explanation may be related to a defective function of IL-30R on monocytes.

We have here provided the first demonstration that IL-27 treatment upregulated HLA-E (but not HLA-G) expression on the cell surface of human monocytes. The upregulation of this immunoregulatory molecule on the latter cells is apparently in contrast with the literature, since different studies have demonstrated a proinflammatory activity of IL-27 on human monocytes [31–33]. However, in the last years, several evidences support the concept that this cytokine may function either as proinflammatory or immunoregulatory factor [34]. In addition, we have very recently demonstrated that IL-27 may act as homeostatic cytokine, limiting the duration and the intensity of adaptive immune response by inhibiting the function of immature dendritic cells [35]. In this view, we may hypothesize that the upregulation of HLA-E on monocytes may function as a negative feedback signal to NK cells, leading to decreased IFN-*γ* secretion by the latter cells that may in turn limit their helper function and consequently the activation of other immune effector cells. In support of this hypothesis, we have previously demonstrated that HLA-E is upregulated in peripheral blood and synovial monocytes from patients affected by juvenile idiopathic arthritis [36], thus suggesting that HLA-E upregulation takes place during autoimmune and inflammatory conditions.

## 5. Conclusions

In conclusion, we delineated a novel potential mechanism of action for IL-27 that may regulate some NK cell functions indirectly through the upregulation of surface HLA-E on human monocytes. Future studies on patients affected by different autoimmune and inflammatory diseases will help to clarify whether this feature may be relevant in physiological and pathological settings.
